# Promiscuous and genome-wide recombination underlies the sequence-discrete species of the SAR11 lineage in the deep ocean

**DOI:** 10.1093/ismejo/wraf072

**Published:** 2025-04-18

**Authors:** Jianshu Zhao, Maria Pachiadaki, Roth E Conrad, Janet K Hatt, Laura A Bristow, Luis M Rodriguez-R, Ramon Rossello-Mora, Frank J Stewart, Konstantinos T Konstantinidis

**Affiliations:** Center for Bioinformatics and Computational Genomics, Georgia Institute of Technology, Atlanta, GA, 30332, United States; School of Biological Sciences, Georgia Institute of Technology, Atlanta, GA, 30332, United States; Woods Hole Oceanographic Institution, Woods Hole, MA, 02543, United States; School of Biological Sciences, Georgia Institute of Technology, Atlanta, GA, 30332, United States; School of Civil and Environmental Engineering, Georgia Institute of Technology, Atlanta, GA 30332, United States; Department of Marine Sciences, University of Gothenburg, Gothenburg, SE-402 34, Sweden; Department of Microbiology and Digital Science Center (DiSC), University of Innsbruck, Innsbruck, 6020, Austria; Mediterranean Institutes for Advanced Studies (IMEDEA, CSIC-UIB), Esporles, 07190, Spain; School of Biological Sciences, Georgia Institute of Technology, Atlanta, GA, 30332, United States; Department of Microbiology & Cell Biology, Montana State University, Bozeman, MT, 59717, United States; Center for Bioinformatics and Computational Genomics, Georgia Institute of Technology, Atlanta, GA, 30332, United States; School of Biological Sciences, Georgia Institute of Technology, Atlanta, GA, 30332, United States; School of Civil and Environmental Engineering, Georgia Institute of Technology, Atlanta, GA 30332, United States

**Keywords:** SAR11, recombination, ANI, species gap, gene sweep, oxygen minimum zone

## Abstract

Surveys of microbial communities (metagenomics) or isolate genomes have revealed sequence-discrete species. That is, members of the same species show >95% average nucleotide identity (ANI) of shared genes among themselves vs. <83% ANI to members of other species while genome pairs showing between 83% and 95% ANI are comparatively rare. In these surveys, aquatic bacteria of the ubiquitous SAR11 clade (Class *Alphaproteobacteria*) are an outlier and often do not exhibit discrete species boundaries, suggesting the potential for alternate modes of genetic differentiation. To explore evolution in SAR11, we analyzed high-quality, single-cell amplified genomes, and companion metagenomes from an oxygen minimum zone in the Eastern Tropical Pacific Ocean, where the SAR11 make up ~20% of the total microbial community. Our results show that SAR11 do form several sequence-discrete species, but their ANI range of discreteness is shifted to lower identities between 86% and 91%, with intra-species ANI ranging between 91% and 100%. Measuring recent gene exchange among these genomes based on a recently developed methodology revealed higher frequency of homologous recombination within compared to between species that affects sequence evolution at least twice as much as diversifying point mutation across the genome. Recombination in SAR11 appears to be more promiscuous compared to other prokaryotic species, likely due to the deletion of universal genes involved in the mismatch repair, and has facilitated the spread of adaptive mutations within the species (gene sweeps), further promoting the high intraspecies diversity observed. Collectively, these results implicate rampant, genome-wide homologous recombination as the mechanism of cohesion for distinct SAR11 species.

## Introduction

Describing how microbial diversity is organized is essential for identifying mechanisms of microbial evolution and predicting the functional consequences of this evolution and diversity. Genomic and metagenomic analyses of both engineered (e.g. bioremediation, wastewater treatment reactors) and natural (e.g. terrestrial or marine) systems have shown that microbial diversity is predominantly organized into “species,” representing tractable clusters of related genomes (reviewed in [1, 2]). Typically, members of such species show ~96%–100% genome-average nucleotide identity (ANI) of shared genes among themselves and are discrete because they show <83% ANI when compared to members of other co-occurring species in the same community [3–5]. More recently, our team observed other discontinuities (or gaps) in ANI values that can be used to define units within a species, most notably genomovars and strains [6, 7]. Specifically, analysis of 330 diverse bacterial species each with at least 10 sequenced representative isolates revealed a scarcity of genome pairs showing 99.2%–99.8% ANI (midpoint at 99.5% ANI) in contrast to genome pairs showing ANI >99.8% or <99.2%, which we suggested to refer to as genomovars [6]. These analyses highlight multiple tiers of genetic organization in microbes, but do not identify the mechanisms that create and sustain this organization.

Several competing hypotheses have been advanced to explain the 95% ANI species or the intraspecies gaps in microbial diversification described above. These include the hypotheses that discrete clusters are maintained by frequent recombination among closely related genomes (*recombinogenic species*) or represent differentiation into separate functional niches (*ecological species*), or combination of these two mechanisms (reviewed in [2, 8, 9]). Recombination for prokaryotes differs fundamentally from sexual reproduction in eukaryotes in that gene exchange or shuffling does not occur during a meiosis step but via vectors of horizontal gene transfer (HGT) followed by recombination of donor DNA into the recipient genome. In eukaryotes, sexual recombination underlies the “biological species concept” resulting in species cohesion. Similar genetic cohesion may also arise in prokaryotes due to recombination, resulting in what we refer to below as “recombinogenic species.” Only homologous recombination involving the replacement of an existing gene/allele by a similar foreign gene is predicted to drive unit cohesion, although homologous recombination could also drive diversification if the recombining partners represent different genomic clusters. Non-homologous recombination brings new genes and potentially new functions into the genome, and thus generally leads to diversification, not cohesion. Also note that it is generally very challenging to define or measure the ecological niche of a microbial taxon, especially in natural settings, and thus directly test for the role of ecological speciation. Accordingly, rejecting recombination as the cohesive force would be sufficient to qualify shared ecology as the presumed mechanism of cohesion. Therefore, we opted to assess the level of recent recombination among the genomes reported here as a force of species cohesion (or lack of cohesion when recombination is low and/or a force of diversification) using an advanced bioinformatic approach that we recently developed for this purpose [10] as well as approaches developed by others earlier (e.g. [11]).

Comparative genomic analysis of major taxonomic groups can be vital for identifying mechanisms of diversification and cohesion in microbes. The SAR11 clade of the *Alphaproteobacteria* class is one of the most ecologically dominant groups on the planet, representing up to half of the total microbial community in the surface or deep ocean [12, 13]. Although the evolutionary origin of the SAR11 cluster remains uncertain [14], phylogenetic analysis of primarily the 16S rRNA gene has indicated that SAR11 bacteria form discrete (sub)clades within the broader SAR11 clade, with these subclades designated by alphanumeric identifiers [15, 16]. Within these well-resolved 16S rRNA gene-based subclades, however, delineating species at the 95% ANI or another threshold has been challenging. This is primarily because mapping metagenomic reads to reference SAR11 isolate genomes or metagenome-assembled genomes (MAGs) has revealed higher intraspecies genome diversity in natural SAR11 populations compared to most other prokaryotic taxa (e.g. ANI values ranging between 90% and 100% ANI) and sometimes indiscrete clusters or species [[3, 17, 18]; and Supplementary Figs S1 and S2]. The SAR11 subclades have therefore been outliers in seeming to lack clear species boundaries, and deviating from the 95% ANI species threshold that commonly works for the great majority of prokaryotic species [6]. These observations raise the possibility of distinct modes of evolution in this major bacterial clade, which we aimed to test here.

Multiple factors have presented a challenge to studying population diversification and cohesion in SAR11. First, reports of indiscrete SAR11 species-level diversity were based on short reads that show larger dispersion of identity values around the mean value (e.g. ANI), making it challenging to infer whole-genome relatedness from these data. Second, few complete or draft genomes exist for SAR11 isolates due to challenges in isolating these bacteria, especially from deep-sea environments [19]. Third, MAGs for SAR11 are typically highly fragmented and incomplete, presumably due to the higher intra- and interspecies diversity that is problematic for genome assembly and binning [20]. Finally, while single-cell amplified genomes (SAGs) can provide valuable genomic data for studying SAR11 diversity, the number of available SAGs from the same or closely related SAR11 subclades is limited [18]. This is again due to a high number of co-occurring species and strains in most samples, and thus not a single subclade has been sampled adequately with typical SAG efforts. That is, a single sample can contain multiple subclades, each represented by several distinct species if based on the conventional 95% ANI standard [20]. Available SAG sequences are also rather incomplete (e.g. <50% completeness) and may suffer contamination issues from co-occurring cells or DNA attached to cells during cell sorting [18, 21]. Therefore, a robust view of SAR11 species-level diversity based on genomes has been elusive to date, especially in sparsely sampled environments including low oxygen regions.

**Figure 1:**
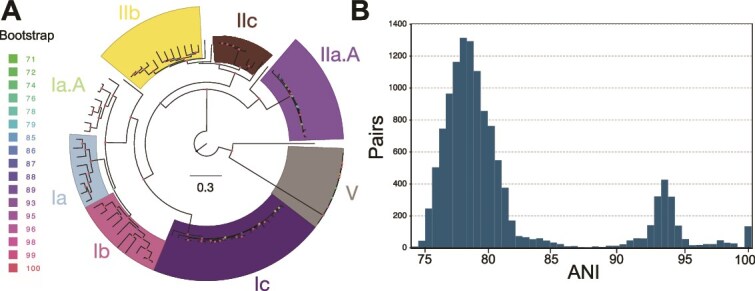
Phylogenetic and ANI diversity of the 105 SAGs used in this study. (A) A phylogenetic tree of the SAGs and selected SAGs and MAGs from the public databases based on a concatenated alignment of 120 universal genes using the GToTree tool [49]. Subclade assignment was determined based on available information for the database genomes. The color on the nodes of the tree indicates the bootstrap support (see legend on the left). (B) ANI distribution between the 105 SAGs used in the study; ANI values were calculated using FastANI (v1.3.3) [25] with default settings. Pairs of SAGs sharing <75% ANI are not shown because such ANI values are not reliable. Note the species gap between 86% and 91% ANI and the structure within the species (>91% ANI) with a peak in data points around 93%–95% ANI.

Studies of SAR11 from the meso- and bathypelagic realms have expanded our understanding of SAR11 diversity and its role in ocean biogeochemistry. All isolated strains of marine SAR11, including members of the ubiquitous *Pelagibacter* genus, are aerobic heterotrophs from surface or near surface waters, adapted for scavenging dissolved organic carbon [15, 19]. Their genomes are small, typically <1.5 Mb, with genomic streamlining as a potential adaptation to the oligotrophic conditions of the open ocean. However, SAR11 subclades can also occur at high abundance in the meso- and bathypelagic [20, 22, 23]. While it has been hypothesized that adaptation in SAR11 does not involve large variations in gene content [15], meso- and bathypelagic subclades have functional genes absent from those in surface SAR11, enabling distinct biogeochemical contributions including potential major roles in organic sulfur cycling [24] and anaerobic pathways of the marine nitrogen cycle [23]. For instance, the SAR11 adapted to the marine oxygen minimum zones (OMZs) have *nar* genes supporting respiratory nitrate reduction in the absence of oxygen. The OMZ SAR11 span at least five subclades, with genomes from four of these subclades (Ic, IIa.A, IIb, and V; Fig. 1A) largely absent from non-OMZ waters but accounting for 10%–30% of the bacterial community at depths with undetectable oxygen [20, 23]. The SAR11 Subclades Ic and IIa are most abundant in OMZs, with the available OMZ-type genomes of Subclade IIa designated as Subclade IIa.A. Subclades Ic and IIa.A in OMZs show 0%–3% intra-subclade differences in 16S rRNA gene sequences and 70%–100% ANI and thus could encompass multiple species based on the frequently used 16S rRNA gene or ANI standards [26]. To provide insights into the species-level diversification process of the OMZ-abundant SAR11 subclades, we report the comparative analysis of 105, high-quality SAGs from 14 samples representing different depths along the oxycline in the Eastern Tropical North Pacific OMZ (see sampling details in Supplementary Table S1 for SAGs and Supplementary Table S2 for metagenomes). Specifically, we (1) test whether these genomes differentiate into discrete genetic clusters, (2) quantify the similarity thresholds that define cluster boundaries, and (3) explore the forces maintaining species clusters, testing whether SAR11 clusters are maintained as cohesive units by recombination. For simplicity, we use “recombination” to refer to homologous recombination below, unless noted otherwise.

## Materials and methods

### Single-cell amplified genome generation

Samples for single cell sorting and genomic sequencing were collected during the oceanographic expedition AT50-08 onboard *R/V Atlantis* at Eastern Tropical North Pacific (ENTP) Ocean. Water samples (see Supplementary Table S1 for sampling stations and coordinates) were prepared by cryopreservation according to the protocol recommended by the Bigelow Single Cell Genomics Center (SCGC). Sorting was performed on 4 April 2023 (within <2 months from the date the first sample was collected) and SAGs were generated with the modified genomic DNA amplification technique, WGA-Y, which enables a substantially improved average genome recovery from single cells (service S-202). In total, 105 SAGs with *C*_p_ values <3 h were randomly selected for sequencing. Genome assembly and draft annotation were performed by SCGC as described in the center’s webpage https://scgc.bigelow.org/capabilities/service-description/.

### Metagenome sequencing and analysis

DNA was extracted from sea water samples following a similar protocol as previously described [23] and detailed in the Supplementary Material (see Supplementary Table S2 for sampling stations and coordinates). Metagenomic coverage was estimated via the Nonpareil software v3.4 (-kmer option) [27]. IDBA-UD was used to assemble short reads into contigs (--min_contig 1000) [28]. Recruitment plots of short reads against MAGs and SAGs were created using the corresponding tool from the enveomics package [29], with minor modifications (see: https://github.com/jianshu93/RecruitmentPlot_blast). Metagenomic genome binning was performed via the binning module of MetaWRAP, which is a wrapper that integrates three widely used binning software MaxBin2, metaBAT2, and CONCOCT [30]. The resulting MAGs were subsequently refined using DASTools (v1.1.2) [31]. Quality control was performed using CheckM [32] while taxonomic classification was obtained via GSearch (v1.3) [33] against the GTDB database v214 [34].

### Recombination analysis

We employed our recently developed bioinformatic pipeline to analyze our SAGs and identify gene exchange/recombination events [10]. Step-by-step details for our main analysis workflow can be found online at: https://github.com/rotheconrad/F100_Prok_Recombination. Briefly, the pipeline identifies reciprocal best matches (RBMs) via BLAST [35] and then uses a metric called *F*_100_ (100% identical RBMs divided by total RBMs) as a proxy for strength of recent gene exchange events. ClonalFrameML was used as an independent method to identity recombination events and estimate recombination to mutation rate (*r*/*m*) based on coalescent models as described previously [36].

Additional details of all methods used in this study are described in the Supplementary Material, including how the SAGs were obtained and processed, how data were processed and quality-checked, and how bioinformatic analyses of the resulting data in terms of phylogenetic tree construction and data visualization were performed. Additional references provide further information about procedures and analytical techniques.

## Results and discussion

### An average nucleotide identity gap does exist for oxygen minimum zone SAR11 species but is shifted

The average completeness and contamination of the 105 SAGs were 73.5% and 0.32%, respectively (Supplementary Table S3); these values are higher than those of most SAG datasets available [18, 21], likely due to recent advancements in the DNA amplification protocol [37]. The 105 SAGs used in this study were assigned to eight subclades based on their core-genome phylogeny (Fig. 1), with the number assigned to each subclade roughly consistent with the relative abundance of the subclade based on metagenome read mapping (Supplementary Figs S1 and S2 and Table S3). For example, the most abundant Subclade Ic in the metagenomes also contained the highest number of SAGs, suggesting that our SAG collection is a representative, random sample of the total SAR11 population *in situ*.

Pairwise comparative analysis of all SAGs revealed a species-level ANI gap (Fig. 1B), but this gap is shifted compared to the great majority of bacterial and archaeal species with adequate numbers of sequenced representatives [6] or other co-occurring species based on metagenomic read recruitment (Supplementary Fig. S1B). Specifically, the ANI gap for OMZ SAR11 SAGs lies between ~86% and ~91%, with the intraspecies ANI values ranging between 91% and 100% and exhibiting a single, prevalent peak at 93%–94% (Fig. 1B). This pattern differs from the 84%–96% ANI gap and 96%–100% intraspecies ANI range reported previously for *Escherichia coli* and other model bacterial species [6]. We observed a similar ANI distribution when the analysis was restricted to the species of two abundant subclades with adequate numbers of SAGs (Subclades Ic and Ib; Supplementary Fig. S3; note that the remaining subclades did not have enough SAGs assigned to them for robust assessment). Therefore, it appears that the OMZ SAR11 community is composed of sequence-discrete species harboring high levels of genomic diversity (Fig. 1B and Supplementary Fig. S2A and B).

### Frequent, recent homologous recombination underlies the SAR11 species

We assessed the level of recent recombination among SAGs using a method recently developed in our lab [10]. Briefly, our approach examines the frequency of identical genes (observed *F*_100_) shared between two genomes relative to the number of such genes expected by chance according to the ANI value (expected *F*_100_), and thus can assess if there is more recombination within vs. between clusters of genomes while normalizing for the degree of relatedness of the genomes being compared [10]. By only focusing on recent exchange events (i.e. identical, or almost identical genes), our approach also circumvents computational challenges associated with historic recombination (e.g. low signal-to-noise ratio due to sequence amelioration to match the mutational biases of the recipient genome) while still being robust for assessing recombinogenic speciation. Further, for recombination to drive species cohesion, it has to be frequent enough (especially compared to the effect of diversifying point mutation) and random across the genome. A non-random (biased) distribution, with recombination spatially restricted to certain genomic regions or concentrated in genes of related function, could indicate selection-driven genetic exchange, mediated by recombination, but not genome introgression, since the non-recombining parts of the genome would continue to diverge.

**Figure 2:**
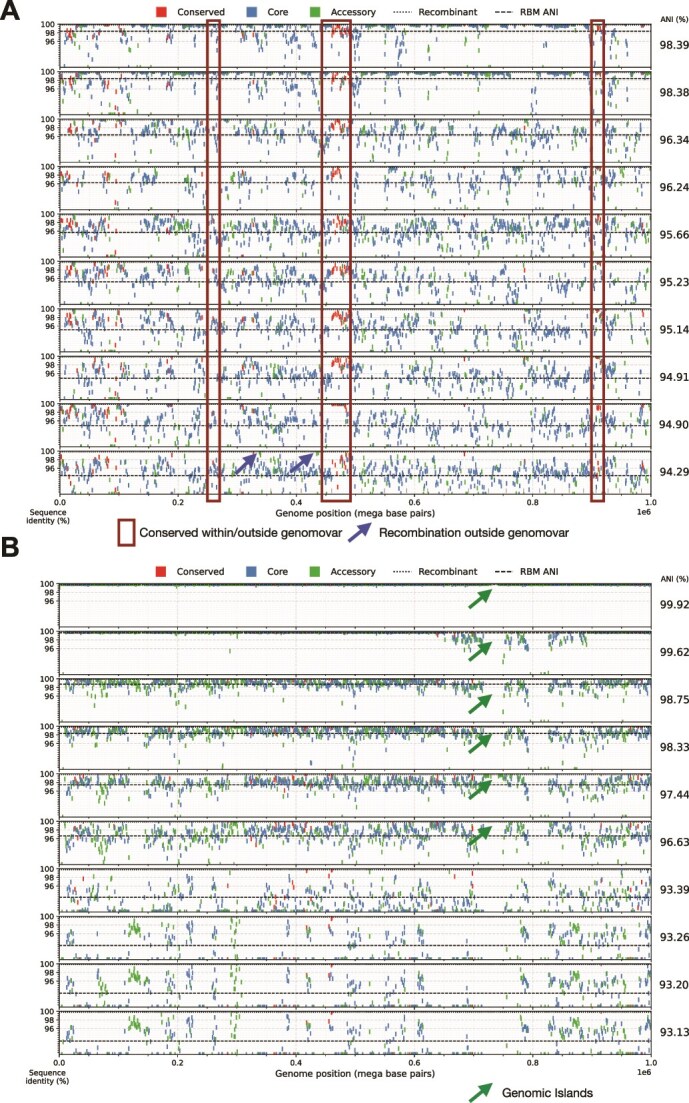
Extensive recent recombination within the SAR11 Subclade Ic and comparison with *E. coli*–*E. fergusonii* genomes. (A) Pairwise RBM genes were identified for 10 SAR11 Subclade Ic SAGs against the same reference, SAG AM-660-D08; (B) similarly, for 10 *E. coli*–*E. coli* (top 6 rows) and *E. coli*–*E. fergusonii* (bottom 4 rows) genomes (reference genome is *E. coli* ASM1374039 from [38]). Each query genome is represented in a different row, and the query genomes are sorted based on decreasing ANI relatedness to the reference (rightmost values). Each rectangular marker represents a gene, colored differently for highly conserved/universal, core, and accessory genes (see figure key). The marker represents the nucleotide sequence identity between the reference and the query genome when the gene is RBM-conserved between the two genomes (*y*-axis) plotted against the position of the gene in the reference genome (*x*-axis). Blue arrows highlight genes that have most likely undergone recent recombination between the reference and the corresponding query genomes as reflected by their high nucleotide identity (>99.8%) compared to the ANI value of the genomes and/or the (lower) identity of the same genes between the reference and another query genome(s), i.e. more related to the reference based on ANI. The green arrows denote a genomic island specific to the reference genome, i.e. not shared by most query genomes and was likely brought into the reference genome by a non-homologous recombination mechanism. The red boxes highlight the highly conserved *narG* and related genes discussed in the text; first box from the left contained *narG* OP-3 type, second box (middle) the *narG* gamma type. See also Supplementary Tables S4 and S5 for details on the genes contained within the boxes.

**Figure 3:**
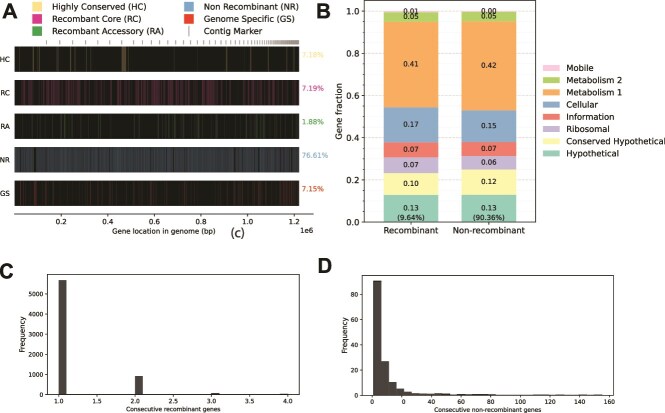
Spatial, functional, and fragment length distribution analysis of recombined segments among the SAR11 SAGs. The figure shows additional analysis of the recombinant genes identified among the 10 genomes of the Subclade Ic shown in Fig. 2A. Specifically, the spatial distribution across the genome, denoted by colored vertical lines, is shown in (A) between the same reference genome used in Fig. 2A (SAG AM-660-D08) and a genome showing ~95% ANI to this reference, separately for non-recombinant and recombinant genes that are core, accessary, or genome-specific (see key on top and two-letter designation on the left). The percentages on the right represent the average fraction of the total genes in the genome that the corresponding gene class makes up (the sum equals to 100%). Functional annotation of recombinant and non-recombinant genes based on the eggNOG-mapper (version 2) is shown in (B). Finally, the length distribution of consecutive (C) recombinant (as a proxy for the length distribution of recombinant fragments) and (D) nonrecombinant genes are shown. Similar results were obtained with other pairs of genomes (not shown).

We first examined the nucleotide sequence identity patterns of individual genes across the whole genome for SAGs of Subclade Ic, the most well-sampled subclade in our collection (*n* = 30 SAGs), in pairwise whole-genome comparisons. We observed that members of the same genomovar are identical or almost identical (nucleotide identity >99.8%) in most of their genes (>50% of the total, typically), except for a few regions (hotspots) that have accumulated substantial sequence diversity (typically 90%–98% nucleotide identity to other members of the same genomovar; Fig. 2, top two genomes). (Note that we used a more relaxed definition for genomovar, defined as genomes sharing >98.5% ANI, compared to our recently proposed definition of 99.5% ANI [6] due to the lack of enough genome pairs showing >95% ANI and the higher intraspecies diversity revealed for SAR11 species in Fig. 1). In almost half of the cases, genes in the diversity hotspots have an identical or almost identical match to another SAG of a different genomovar in our collection, indicating recent HGT mediated by homologous recombination from that genomovar or its recent ancestors (Fig. 2, red boxes; and Fig. 3A for a summary picture). It is thus likely that the other half of the genes in the diversity hotspots are also the product of recent HGT, but we did not have the donor genome within our SAG collection to confirm the HGT event (e.g. identify the high-identity match). Most of the genes in these hotspots represented core genes shared among the species, although several accessory (or variable) genes were also noted. Alternatively, these divergent genes could represent regions of hypermutation, but this scenario is less likely given the high identity across the rest (majority) of the genome and that the predicted functions of the divergent genes appear to be a random subsampling of the functions in the genome as a whole (Fig. 3A). Hence, the hotspots of sequence diversity between members of the same genomovar are unlikely to represent hypermutation or positive (adaptive) selection (see also next section for a probable case of positive selection).

**Figure 4:**
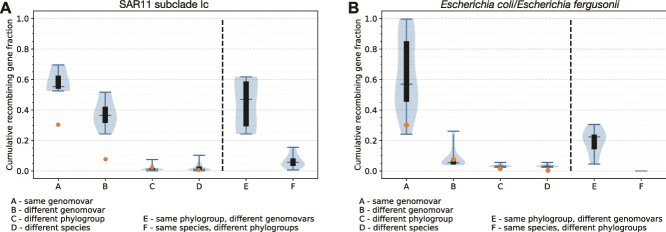
Fraction of identical, or nearly identical, genes a genome shares with all other genomes within or between genomovar, phylogroup, and species for SAR11 Subclade Ic (A) and *E. coli* (B). Each genome was compared to all other genomes within each grouping (A–F) and the cumulative fraction of shared identical genes (identity >99.8%) was recorded and plotted using the custom script *Allv_RBM_Violinplot.Py*. Note that an individual gene is counted only once for this analysis so as not to overestimate recombination regardless of how many genomes were found to have recently exchanged the gene. The groupings were as follows: (A) genomes within the same genomovar defined at >98.5% ANI, (B) genomes in each separate genomovar within the same phylogroup, excluding genomes from the same genomovar, defined at 98.5% > ANI > 96.5%, (C) genomes in each separate genomovar within different phylogroups defined at 96.5% > ANI > 93.5%, and (D) genomes of the other species for *E. coli* (i.e. *E. fergusonii* vs. *E. coli*) or the most divergent SAGs of the same species for SAR11, defined at 93.5% > ANI > 91%. Groups A through D show comparisons between one reference genome against all available genomes in a single genomovar of the corresponding group (e.g. a genomovar from the same phylogroup as the reference for B except the genomovar that the reference is assigned to). Groups E and F show comparisons between one reference genome against all available genomes in all genomovars of the corresponding group (e.g. all genomovars available from the same phylogroup as the reference for E except the genomovar that the reference is assigned to). Data are presented in hybrid violin plots where the top and bottom whiskers show the minimum and maximum values, the middle whisker shows the median value, the black boxes show the interquartile range, and the light blue regions show the density of values along the *y*-axis. Data are based on the 30 SAGs of SAR11 Ic subclade of this study and 20 *E. coli* and 10 *E. fergusonii* published previously [38]. We used a similar number of genomes in order for the results to be directly comparable between the two taxa. Note that while one or a few genomes create extreme outliers, overall, the fraction of identical genes gradually decreases among more divergent genomes compared ( A→ D). Also, note that our modeling analysis (orange circles on the graph; see main text for more details) suggests, e.g. that only about 6%–7% of the total genes in the genome should be expected to be identical or nearly identical among genomes showing around 98% ANI if there is no recent recombination (i.e. the B and E groups). All SAR11 and several of the *E. coli* data points represent many more such genes in the one-to-one genomovar (Group B) or one-to-many genomovars (Group E) comparisons at this level, revealing extensive recent gene exchange. Finally, note that the amount of nearly identical genes for some groups of *E. coli* (e.g. B) may look lower compared to our previous publication that included the same genomes because the latter publication used the full dataset of genomes available (*n* = 342).

To further quantify recombination, we counted all genes identified as recently recombined between any two genomes by our approach as described previously [10]. That is, these genes should show 99.8%–100% nucleotide sequence identity between genomes of different genomovars (i.e. ANI <98.5%) and not be highly conserved—at the sequence level—due to functional constraints such as the examples shown in Fig. 2 and contrasting with genes like the 16S rRNA gene that evolves slowly, under strong functional constraints. The length of the presumed recombined segments, using the total length of consecutive recombined genes as a proxy, was found to be similar to that observed in previous laboratory recombination studies [39] and to range between 1 and 20 kb, with the majority being 1–3 kb (Fig. 3C). Our spatial analysis also showed that there were no regions in the genome longer than 100–200 kb, which were free of recombination in at least some pairs of genomes (see Fig. 3A for an example). Further, while the fraction of the genome affected by recombination between any two genomes (of different genomovars) was almost always <20% of total genome length, when we compared one reference genome against representative genomes of all available genomovars (other than its own genomovar) and summed all recombination events (with all possible partners in the analysis), the genomic fraction affected by recombination often approached 40% or higher (Fig. 4, B and E groups). Such results were obtained with all reference genomes and were not specific to any specific genome of Subclade Ic.

Using the same approach for detecting and quantifying recombination, we recently showed that genomes of *Salinibacter ruber*, an environmental halophilic bacterium, and *E. coli*, the enteric model bacterium and opportunistic pathogen, frequently engage in unbiased homologous recombination that has five or more times the effect of diversifying mutation on sequence evolution (Supplementary Fig. S4B and in [10]). Accordingly, recombination is responsible for maintaining species- and intraspecies sequence-clusters (units) for these two species [10]. The majority of the SAR11 SAGs of the same species of Subclade Ic (i.e. sharing >91% ANI) showed levels of homologous recombination similar or even higher than those observed previously for *E. coli* (Figs 2, 4, and Supplementary Fig. S4A). For instance, in comparisons of one reference SAR11 genome against all genomes of a different genomovar showing between 96.5% and 98.5% ANI to the reference (i.e. one against a whole, narrow lineage; Fig. 4, B groups), the fraction of genes identified as recently exchanged was, on average, 35% of the total genes in the genome, whereas this percentage ranged between 5% and 25% for *E. coli* genomes with comparable ANI, and was 6% in the absence of any recombination (expected *F*_100_). Our previous study of the same *E. coli* genomes using an empirical approach to estimate the ratio of mutations purged by homologous recombination (*r*) vs. mutations created by point mutation over the same period of time (*m*), or simply the *r*/*m* ratio, revealed that the level of recombination observed for these *E. coli* genomes translates to an *r*/*m* ratio higher than 1 and often around 3–5 for several genome pairs [10]. The SAGs analyzed here appear to have similar, if not higher, rates of recombination than the *E. coli* genomes, especially among genomes with ANI between 96% and 98% (Fig. 4 and Supplementary Fig. S4). Similar *r*/*m* ratios were also obtained with the independent approach of ClonalFrameML (Supplementary Fig. S5) [11, 36]. Therefore, it appears that these SAR11 SAGs are engaging in genome-wide, rapid recombination that affects sequence identity much more than diversifying (point) mutations, revealing a recombinogenic rather than clonal pattern of sequence evolution.

Substantial recent recombination was not only observed among highly related SAR11 genomes (e.g. showing >96% ANI) but also among several—but not all—genome pairs related at 91%–94% ANI, albeit the measured recombination frequency was comparatively lower for the more divergent genomes (Figs 2 and 4). It was not possible to compare these results to values for *E. coli*, given that all genomes classified as *E. coli* share >95% ANI. However, *Escherichia fergusonii* shares ~91%–94% ANI with *E. coli*, a level comparable to that observed among the most divergent SAR11 SAGs of the same species. The *E. fergusonii* genomes analyzed here originated from the same geographic site in the United Kingdom (100 km radius) as the *E. coli* genomes [38], and thus could have engaged in more genetic exchange in comparison to genomes drawn randomly from the public databases. Nonetheless, comparing *E. fergusonii to E. coli* revealed a few hotspots of recombination, but these appear to be located in the exact same regions (genomic islands) in the available *E. fergusonii* genomes (Fig. 2A, bottom rows). Therefore, these recombination events most likely reflect selection-driven recombination or are restricted to genomic islands and are unlikely to lead to genome introgression, even if the estimated *r*/*m* ratio is >1 due to these events. In contrast, recombination in the SAR11 genomes sharing 91%–94% ANI occurs in many genes distributed in different regions for different query genomes, suggesting unbiased recombination (Fig. 2A, bottom rows; note the multiple high-identity genes found across the genome for SAR11 genomes but not for the *E. coli* vs. *E. fergusonii* comparison; and Fig. 4, D and F groups).

Collectively, it appears that for the SAR11 Subclade Ic species evaluated here, homologous recombination is frequent and random enough (spatially across the genome) and likely sufficient to serve as the mechanism for species cohesiveness. While these results are predominantly based on Subclade Ic for which we had the highest number of SAGs, similar frequency of recombined genes and a lack of spatial bias across the genome were observed for SAGs representing the surface (non-OMZ) (Supplementary Fig. S6), indicating these patterns may be broadly applicable across SAR11 species from distinct oceanic niches.

### Gene sweeps, rather than genome sweeps, shape SAR11 functional diversity, consistent with recombinogenic species and high intraspecies diversity: the case of nitrate reductase

While recombination affected almost every gene in the genome (Fig. 2A), we also observed three hotspots where recombination was more frequent than in the rest of the genome and consequently resulted in low sequence divergence of the affected genes (Fig. 2A). Two of the hot spots encompassed genes encoding the two variants (OP3 and Gamma variants) of the respiratory nitrate reductase (NarG) previously identified as distinguishing OMZ SAR11 subclades from their counterparts in oxic water columns [23]. The third hotspot included genes of other metabolic functions (Supplementary Table S4). The first hotspot encompassed only the *narG* gene encoding the OP3 nitrate reductase variant, while the second hotspot included the entire operon (*narGHIJ*; Supplementary Table S5) encoding the Gamma NarG variant. The OP3 and Gamma *narG* variants share only about ~45% amino acid identity (no detectable identity or alignment at the nucleotide level), and therefore are unlikely to undergo homologous recombination with each other. The genes in the two *nar*-encoding hotspots showed very high sequence identity across SAGs, ranging from ~99% to 100%, even among SAGs that show 93%–94% total genome ANI. A plot showing metagenomic reads recruited to the contig containing the *narGHIJ* operon confirmed that the operon exhibits consistent levels of high similarity across the *in situ* SAR11 community (Supplementary Fig. S7), not only among our SAGs.

**Figure 5:**
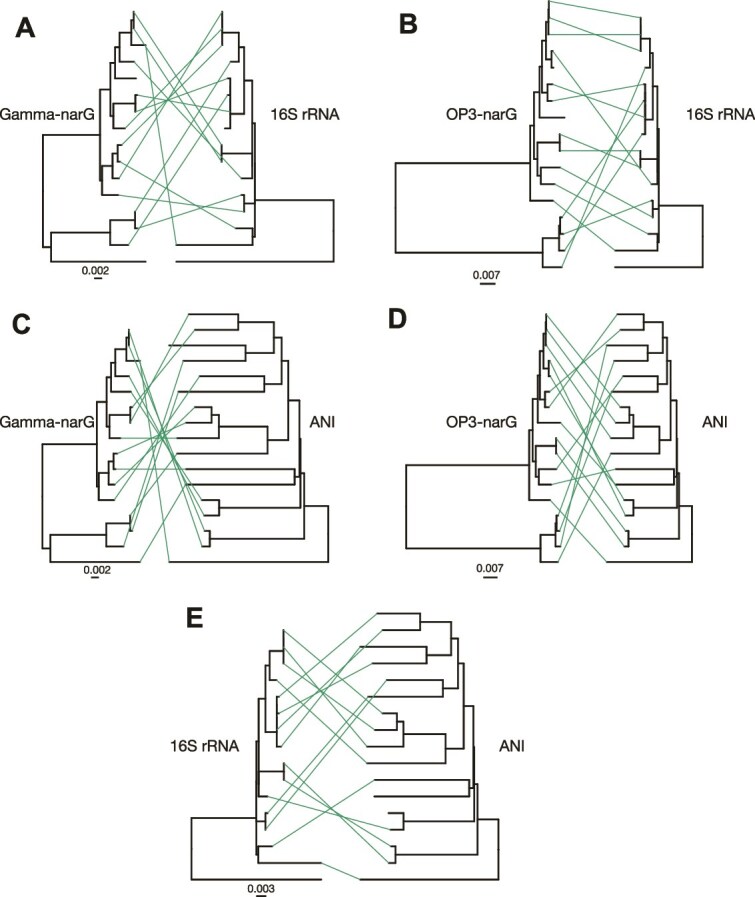
Phylogenetic relationships among Subclade Ic SAGs based on ANI, 16S rRNA, and *narG* genes. See labels on top of each tree for which gene (or genome ANI) the tree represents. In short, the top row represents (A) gamma-type *narG* (second hot spot in Fig. 2A) and (B) OP3-type *narG* (first hot spot in Fig. 2A) in comparison to the 16S rRNA gene, whereas the second row (C and D) shows the same two *narG* gene trees as in (A and B) in comparison to an ANI tree. The last row shows the 16S rRNA gene in comparison to the ANI tree (E). The scale bars denote the substitution rate. All trees are built using nucleotide sequences and the neighbor-joining algorithm. The long, unpaired branch in 16S rRNA and *narG* trees represents Subclade V and was used as the outgroup. All trees are midpoint rooted. Note the similar level of sequence diversity within the *narG* and the 16S rRNA genes and that several cases of probable horizontal gene transfer of these genes can be observed when taking the ANI as the species tree (e.g. lines connecting tree branches represent different topologies compared to the ANI tree).

This level of high sequence identity (>99%) in *nar* genes is comparable to only that for rRNA genes, which are known to be highly conserved due to functional constraints (Fig. 5 and Supplementary Table S5). Such high similarity is extremely rare for functional genes such as *nar* [40], unless the genes are recently horizontally exchanged or the genomes are very closely related (e.g. >99% ANI; thus, not enough evolutionary time for sequence diversification), which is not the case for most SAR11 SAGs analyzed here. The phylogenies of both *narG* variants were not congruent with that based on corresponding 16S rRNA gene sequences, further suggesting HGT. In fact, when we compared the *narG* and 16S rRNA gene phylogenies against an ANI-based or a core-genome tree, we observed at least two cases of clear HGT of not only *narG* but also the 16S rRNA gene within (but not outside) Subclade Ic, assuming the ANI tree best represents the species phylogeny (Fig. 5). These results are consistent with the rampant and unbiased recombination described above, which is notable for the rRNA genes that are thought to be transferred horizontally only rarely [41]. Collectively, these results suggest that *narG*, and likely the entire *nar* operon, has undergone a gene sweep event within the Subclade Ic species, presumably caused by positive (adaptive) selection for the prevailing *narG* allele (allele is defined here as sequences of the gene showing >98.5% nucleotide sequence identity; Supplementary Table S5). While frequent recombination has seemingly kept *nar* sequence diversity low across the genomes, the apparent selective advantage of *nar* has not caused sweep events at the level of the genome–genomes with *nar* did not outcompete and drive extinct genomes without *nar*. If a genome sweep event had taken place, the remainder of the genome would be expected to show low sequence divergence similar to that of the *nar* operon (i.e. >99% ANI vs. 91%–100% ANI observed, with the median around 94%). Previous research has shown nitrate reduction, mediated by Nar, to be a key energy-generating pathway for OMZ-associated SAR11 across all OMZ SAR11 subclades [23], consistent with the presumed strong positive selection for Nar function.

### Deep-sea SAR11 lack genes encoding several proteins involved in the sequence fidelity of the homologous recombination apparatus, which further promotes the high intraspecies diversity

Previous experimental studies have shown that the frequency of recombination drops substantially around 95% nucleotide identity albeit recombination is still possible even among pieces of DNA that are <90% identical, depending on the secondary structure of the recombining DNA and the promiscuity of the corresponding enzyme systems, among other factors [42]. More recently, high-throughput transformation studies at the whole-genome level have further corroborated these earlier findings and showed that homologous recombination efficiency in *Bacillus subtilis* drops by 5-fold or more for sequences that share 94%–95% nucleotide identity relative to those that share 99%–100% identity [39]. This prior work has also shown that homologous recombination efficiency drops to almost zero for sequences showing around 88%–90% identity (~20-fold reduction compared to 99%–100% identical sequences), which corresponds to the highest intraspecies divergence level observed for the SAR11 genomes studied here (91%–100% ANI; if the ANI is 91%, this would mean that several individual genes in the genome show 88%–91% nucleotide identity). There are two additional lines of evidence that further corroborate that SAR11 bacteria are able to recombine even sequences that are around 90% nucleotide identity. First, all genomes have a least a few protein-coding genes with a 99%–100% nucleotide identity match with a second genome that shares around 94% ANI in the rest of the (shared) genes (i.e. a genome representing a divergent genome, but of the same species), which represents direct evidence of recent exchange of these genes with the latter genome or its immediate ancestors (Fig. 2). Second, when we examined the underlying identity distribution of shared genes between two genomes showing high ANI (98.5%), we detected several genes with lower nucleotide identities of ~90%–92%. Such genes made up a larger fraction of the total genes in the genome in SAR11 when compared to *E. coli* genomes of similar ANI, causing the distribution to deviate further from a normal distribution around the mean value (the ANI value; Supplementary Fig. S8). It is unlikely that these represent genes with an increased rate of fixed mutations (increased mutation rate) for the reasons mentioned above but also because several of these genes encode core functions. Rather, a more parsimonious scenario is that such genes are the products of recombination involving the most divergent members of the species.

It is intriguing that SAR11 shows higher intraspecies diversity compared to other bacterial species. This could be due to a relatively high amount of evolutionary time since the last genome sweep event but also to higher recombination promiscuity, notably if the identity threshold for homologous recombination is comparatively low in SAR11. Consistent with the latter interpretation, OMZ SAR11 species, similar to their surface *Ca. Pelagibacter ubique* relatives, are remarkably different from the other alphaproteobacterial species in the repertoire of their genes for homologous recombination and repair. Specifically, genes encoding the RecBCD complex, which initiates homologous recombination by helping to load the primary homologous recombination protein RecA onto double-stranded gaps, are absent from both SAR11 Ic and IIa.A representatives. Similarly, among *Alphaproteobacteria*, *Ca. Pelagibacter ubique* and Subclade Ic are unique in that they lack the RecF pathway for homologous recombination associated with DNA repair (Supplementary Fig. S9), suggesting that these bacteria might utilize the recently proposed RecF-independent pathway, the RecOR pathway, for the loading of RecA for single-strand gap repair. It is hypothesized that the simplified RecOR pathway might initiate homologous recombination at both single-stranded and double-stranded breaks (reviewed in [43]), potentially resulting in a sequence identity threshold for recombination different from that of other *Alphaproteobacteria*. Further and perhaps more importantly, two additional key genes involved in the mismatch repair system—*mutS* and *mutL*—are missing from the SAGs of Subclades Ic and IIa.A (Supplementary Fig. S9), despite their presence in all other *Alphaproteobacteria* genomes. The MutSL protein complex corrects base pair mismatches and acts as an anti-recombinant by preventing homologous recombination of slightly diverging sequences [43]. Collectively, these results indicate that SAR11 species may be evolving under enhanced rates of nucleotide substitutions and more promiscuous recombination, which could account, at least partially, for the higher diversity of ANI values observed within species (e.g. Fig. 1A and B). It would be interesting to further test this hypothesis in the future using experimental studies. Such experimental studies could, e.g. involve transformation rates of DNA of varied sequence identities compared to the recipient genome as performed recently for *B. subtilis* [39]. A major limitation in performing such studies of SAR11 bacteria is the lack of representative isolates of the abundant deep-sea SAR11 subclades with robust growth in laboratory media and conditions, but new approaches to cultivation [19] may soon yield appropriate isolates for these purposes.

The intraspecies genetic structure observed for SAR11 might also be due, at least partly, to a long period of time since the last diversity-purging selection event, in addition to the lack of proofreading proteins mentioned above. Members of the species could have been evolving together since this event, accumulating point mutations but also engaging in frequent homologous recombination. The fact that most SAGs share 93%–95% ANI and only a few share >96% (Fig. 1B) is consistent with this scenario of a long time since the last population/genome sweep and frequent recombination between relative divergent genomes showing 93%–95% ANI. (It is also important to recognize that 1% nucleotide difference [corresponding to 99% ANI] represents thousands of generations [and likely many years] since the last common ancestor, in the absence of recombination [44].) It follows that genomes that accumulate additional mutations beyond this species boundary (i.e. they diverge below the 91%–92% ANI threshold) could either be driven extinct by competition with other members of the species or diverge to represent distinct species, potentially accounting for the 86%–91% ANI gap (Fig. 1). The former scenario of being outcompeted could be driven by a lack of recombination with the remaining members of the species. Such lack of recombination could prevent the acquisition of advantageous alleles or render the cells unable to get rid of disadvantageous alleles, resulting in cells at a significant disadvantage. The results involving nitrate reduction-encoding *nar*, with all members of the 91%–100% ANI species cluster containing highly identical *narG* sequences (>97.5% nucleotide identity; Supplementary Table S5), and even SAGs from different subclades showing high *nar* relatedness (Supplementary Fig. S10), is compatible with our interpretation in the latter scenario. Any OMZ SAR11 genome unable to acquire these—apparently advantageous—*nar* alleles—likely via homologous recombination—is at a serious disadvantage under this scenario, highlighting the importance of nitrate respiration in these OMZ-adapted SAR11 lineages.

### Conclusions and perspectives

The SAG sequences analyzed here corroborate previous results [3, 20], indicating that OMZ SAR11 populations are organized in sequence-discrete species. Further, we show that these populations have a wider range of intraspecies diversity than most other bacteria studied to date by more precisely quantifying this diversity and showing that the intraspecies ANI values for the OMZ SAR11 range between 91% and 100% vs. 96% and 100% for other species [6]. Note that we do not favor the splitting of SAR11 species at higher ANI values, even though there appears to be fewer SAG pairs showing 96%–99% vs. 93%–94% ANI (Fig. 1B), because we still observe frequent recombination among the latter SAGs, and the lack of genome pairs is much more pronounced in the 86%–91% ANI range (compared to 96%–99%), as well as for practical reasons (e.g. too many resulting species). Our results also explain several of the challenges reported in the recent literature for those studying species-level diversity in the SAR11. That is, the wide range of intraspecies sequence diversity in SAR11 (Fig. 1B) is outside the range of identity that most assembly programs typically use to merge sequences (i.e. at least 97%–98% identity), which presumably accounts—in part—for the highly incomplete SAR11 MAGs recovered previously [20, 45]. Further, the pattern of indiscrete species observed by several previous studies (e.g. [15, 46]) is largely due to artifacts of the short-read sequence analysis. That is, short reads show larger dispersion around the mean nucleotide identity value (e.g. ANI) when Single Nucleotide Polymorphisms (SNPs) accumulate over relatively short pieces of DNA, which, given the level of sequence diversity revealed among our SAGs, is rather common within SAR11 species. Further, our analysis revealed that homologous recombination may be the force driving species cohesion for these genomes as recombination was found to be both frequent (i.e. greater impact on sequence evolution compared to diversifying mutation) and random across the genome. In contrast, non-homologous recombination that could bring new functions into the genome was at least 10 times less frequent among the SAGs (Fig. 2), suggesting that ecological speciation is less important for species cohesion (but not necessarily for species diversification; see below) compared to recombinogenic speciation. High rates of recombination among the SAR11 genomes have been noted previously in several studies [47, 48], consistent with some of the results reported here. However, these previous studies did not link recombination to the species and intraspecies units revealed here. Moreover, the previous studies have not shown that recombination is random (unbiased) across the genome (as opposed to selection-driven, and thus spatially and functionally biased) and frequent enough to serve as a cohesive force for the SAR11 species as we have performed here (e.g. Figs 2 and 4). Our gene content analysis also revealed a potential mechanism (i.e. lack of sequence proofreading proteins) that could explain the apparent high frequency of homologous recombination even among several members of the species that show sequence relatedness near the lowest limit at which homologous recombination is still possible (i.e. around 90% nucleotide identity).

In summary, our results show that while the SAR11 clade is an outlier with respect to its level of intraspecies genome diversity, this major bacterial group does diverge via many sequence-discrete species. The ANI threshold delimiting these species is notably lower (91%–92%) than that observed for most other major bacterial lineages studied to date (95%–96%). The cohesive force for speciation in SAR11 appears to be homologous recombination, and the higher intraspecies diversity appears due—at least in part—to the SAR11 species being more promiscuous, with recombination commonly occurring between genomes sharing relatively low sequence identity. This promiscuity could be mechanistically attributable to a unique recombination apparatus in SAR11 compared to other *Alphaproteobacteria*, although this hypothesis would require further experimental validation. How closely related, yet distinct SAR11 species co-occur—as opposed to one outcompeting the other—in close proximity within the OMZ remains to be evaluated in the future. The co-occurrence may be facilitated by different substrate affinities of their *NarG* operon—and recombination-mediated *NarG* allele homogenization within but not in-between species as explained above—or other functional and/or ecological differentiation mediated by their substantial species-specific gene content [20]. The results presented here, and the bioinformatic methodology used to identify and track the species units, provide an important foundation, e.g. the units to study, to tackle these questions in future ecological studies.

## Supplementary Material

SAR11_recombination_Supplementary-Material-F_wraf072

## Data Availability

Metagenomic raw data can be found in NCBI (project accession number PRJNA1124864). Raw sequence of single amplified genomes can be found in NCBI (PRJNA1124867). Our recombination detection pipeline is available at: https://github.com/rotheconrad/F100_Prok_Recombination and 10.5281/zenodo.13922077.
