# Supplementary material for: Promiscuous and genome-wide recombination underlies the sequence-discrete species of the SAR11 lineage in the deep ocean
Source: ISME J. 2025 Apr 18;19(1):wraf072. doi: 10.1093/ismejo/wraf072 (PMC12374537; doi:10.1093/ismejo/wraf072)
Supplement: SAR11_recombination_Supplementary-Material-F_wraf072 [file sar11_recombination_supplementary-material-f_wraf072.pdf]

Supplementary Material

Complete Material and Methods

Collection of ETNP sea water samples

Samples for metagenomic sequencing were collected from the ETNP OMZ (R/V Sally Ride (SR2114), December 2021 – January 2022). Sea water for metagenomes was collected from all nine depths for all five sampling stations (See Table S2 for details). Collections were made using Niskin bottles on a rosette containing a conductivity–temperature–depth profiler (Sea-Bird SBE 911plus), as described previously [1].

Water sampling and Single Cell Amplified Genome (SAG) generation

Samples for single cell sorting and genomic sequencing were collected during the oceanographic expedition AT50-08 onboard *R/V Atlantis* at Eastern Tropical North Pacific (ENTP) Ocean. Sea water for single-cell sorting and single amplified genome (SAG) analysis was collected from two stations. Station coordinates and collection dates of each sample are described in Table S1. Water samples were prepared by cryopreservation according to the protocol recommended by the Bigelow Single Cell Genomics Center (see details below). Briefly, triplicate 1 ml samples of bulk sea water (no prefiltration) were gently mixed with 100 µl of a glycerol TE stock solution (20 ml 100× TE pH 8.0, 60 ml sterile water, 100 ml glycerol) and frozen at -80 °C. Water was sampled from the Niskin bottles immediately upon recovery, and 1ml subsamples were transferred to cryovials. For water samples from low oxygen concentrations or water samples in which oxygen was undetectable (all samples besides the one recovered from 1000m), the transfer was performed within an anaerobic glove bag. Additionally, the cryovials used had been stored in glass bottles filled with helium gas to off-gas oxygen. To select for viable cells or cell with respiratory activity, the subsamples were amended with 1µL BacLight™ RedoxSensor™ Green (Thermofisher Scientific) following the protocols of the Single Cell Genomics Facility (SCGC), Maine, USA and Lindsay et al [2]. After short vortexing (1-2 secs), the vials were incubated in the dark at in situ temperatures for 30 mins. Following incubation 100ml of glyTE buffer was added (5% glycerol, 10 mM Tris, and 0.1 mM EDTA final concentration). Vials were frozen at -80˚C and shipped to the SCGC for sorting. Sorting was performed on April 4th, 2023 (within less than two months from the date the first sample was collected) and SAGs were generated with the modified genomic DNA amplification technique, WGA-Y, which enables a substantially improved average genome recovery from single cells (service S-202). Around 100 SAGs with Cp values less than 3h were randomly selected for sequencing. Genome assembly and draft annotation were performed by SCGC as described in the center's webpage https://scgc.bigelow.org/capabilities/service-description/. Briefly, SAG paired-end libraries were created with Nextera XT kits (Illumina), sequenced with a NextSeq 500 (Illumina) and de novo assembled using a workflow utilizing SPAdes [3], as previously described [4]. The total number of SAGs assembled was 1152, 128 of which were affiliated to *Ca.* Pelagibacterales using GSearch (v1.3.0) against GTDB database v214.

Metagenomic sequencing and analysis

DNA was extracted from sea water samples following a similar protocol as previously described [1]. Briefly, DNA was extracted from biomass on collecting filters using the MoBio Power Soil kit (MoBio Inc. Carlsbad, CA, USA) and libraries were prepared for metagenomic sequencing using the Illumina DNA library prep kit with unique dual indexing according to manufacturer's instructions, except that the protocol was terminated after isolation of cleaned double stranded libraries. An equimolar mixture of the libraries was

sequenced on an Illumina NovaSeq 6000 instrument at the Molecular Evolution Core, Georgia Institute of Technology. A negative control was used to make sure that our samples are not contaminated during the extraction process. Initial adapter trimming and demultiplexing of sequenced samples was carried out by the instrument. Adapter trimming and quality control of short reads was performed via fastp and then checked via Falco via MiGA pipeline [5]. Metagenomic coverage was estimated via the Nonpareil software v3.4 (-kmer option) [6]. IDBA-UD was then used to assemble short reads into contigs (--min_contig 1000) [7]. Recruitment plot of short reads against MAGs and SAGs were created using the corresponding tool from the enveomics package [8], which minor modifications (see: https://github.com/jianshu93/RecruitmentPlot_blast). Metagenomic binning was then performed via the binning module of MetaWRAP, which is a wrapper that integrates three widely used binning software MaxBin2, metaBAT2 and CONCOCT [9]. MAGs/bins from the binning module were then refined using DASTools (v1.1.2) [10]. Quality control was performed using CheckM [11] while taxonomic classification was obtained via GSearch (v1.3) [12] against the GTDB database v214 [13].

Recombination analysis

We employed our recently developed bioinformatic pipeline to analyze our SAGs and identify gene exchange/recombination events. Step by step details for our main analysis workflow can be found online at: https://github.com/rotheconrad/F100_Prok_Recombination. Briefly, the pipeline identifies reciprocal best matches (RMBs) via BLAST [14] and then uses a metric called F100 (100% identical RBMs divided by total RBMs) as a proxy for strength of recent gene exchange events. ClonalFrameML was used as an independent method to identity recombination events and estimate recombination to mutation rate (r/m) based on coalescent models as described previously [15].

Phylogenetic analysis and functional annotation

SAG genome trees were built using either GToTree (based on a concatenated universal gene alignment) [16] or ANI distance matrices produced by FastANI [17], while gene trees were built via IQ-TREE2 [18] after aligning the corresponding sequences with MUSCLE [19]. Genes were extracted from SAGs using protein BLAST and annotated with eggNOG-mapper (v2.1.12) [20] against the eggNOG Database (v6.0) [21].

Data visualization

All plots were obtained via R ggplot2 library (v3.4.4) or python Matplotlib library (v3.9.0).

Supplementary Figures

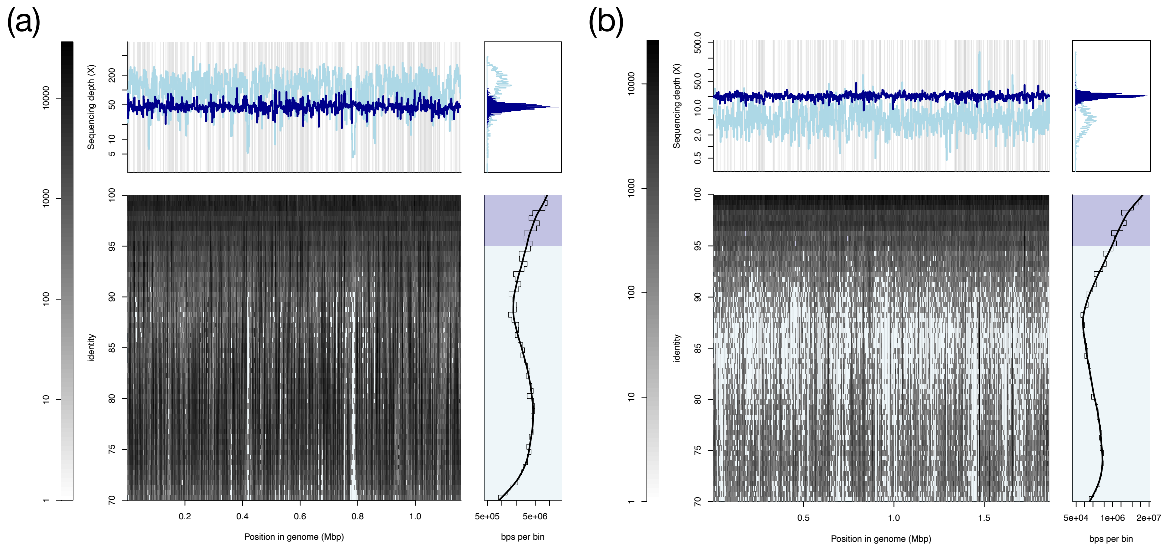

Figure S1. **Recruitment plot for (a) a SAR11 MAG (subclade Ic) and (b) an *Acidimicrobiales* sp. MAG against the same metagenomic sample from station 38, depth 302 m from which the MAGs originated**. The SAR11 MAG quality was estimated to be 89.5% complete and 13.2% contaminated. The main panel of (a, bottom center) shows the position (x-axis) and identity (y-axis) of the metagenomic reads that map on the reference MAGs, which are summarized into two categories: reads above 95% (dark blue) and below 95% (light blue) sequence identity. These two categories are maintained in the other panels. The lower - right panel shows total reads mapped (in base pairs, log scale) for different identities across the entire genome while the upper panels show the coverage of MAGs by reads at each position across their sequence. Note that the sequencing coverage is in log scale. Note the clear, sequence-discrete population for the *Acidimicrobiales* sp. around 80% to 95% nucleotide identity, but the much less discrete SAR11 population. See reference [8] for further details on the recruitment plots.

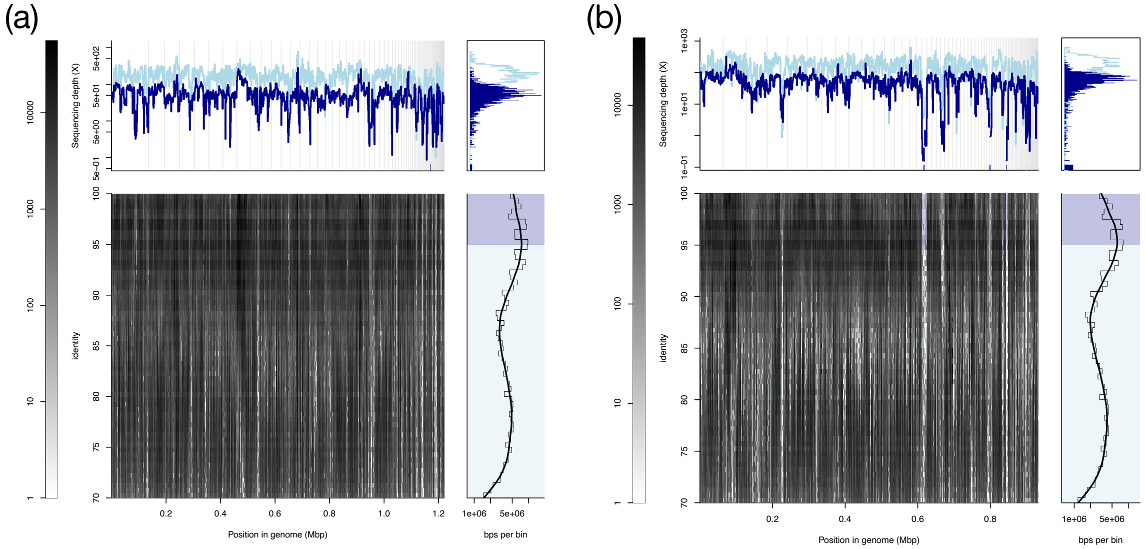

Figure S2. **Recruitment plot of a SAR11 SAG of subclade (a) Ic and (b) IIa.A against the same metagenomic sample from station 38, depth 302 m**. Figure S2 is identical to Figure S1 except that the best quality SAG for each subclade was chosen as a reference genome for mapping of the metagenomic reads. Note that the peak in reads mapping around 95% is consistent with the ANI distribution revealed when comparing all SAGs (i.e., Figure 1), and that the total population is rather indiscrete, especially in comparison to Figure S1(b), although a weak valley can be observed around 90% nucleotide identity (similar to Figure 1b based on whole SAG sequences).

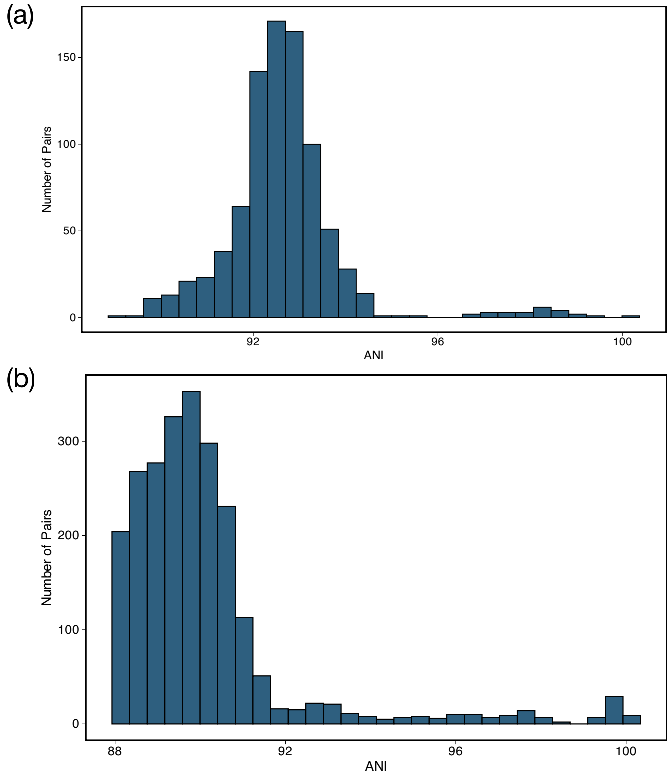

Figure S3. ANI distribution for SAR11 (a) subclade Ic SAGs and (b) Ib. A total of 30 SAGs were used that were assigned to subclade Ic (870 comparisons in total). ANI was calculated using FastANI (v1.3.3). The difference with Figure 1b is that it shows all the SAGs from all the clades from the OMZ. Subclade Ib (surface clade) SAGs were obtained from reference [22], a total of 80 SAGs (6320 pairs in total) from the same sampling site (BAT) were used, with genome pairs showing ANI <88% not shown. Note that the ANI gap is not as pronounced in the surface subclade Ib and this is due, at least in part, to the lower quality of the SAGs that were sequenced based on earlier protocol [22].

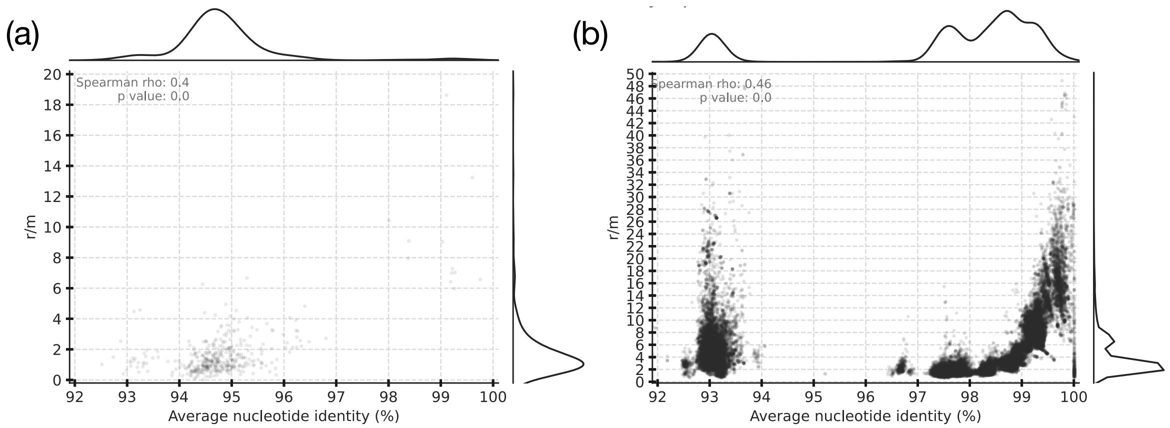

Figure S4. Recombination to mutation ratio (r/m) versus ANI for (a) SAR11 subclade Ic and (b) *E. coli*. See Methods & Materials for how r/m was calculated. The r/m ratio (y-axes) was estimated for all genome pairs in our collection for each species and is plotted against the ANI value of the genome pair compared (x-axes). The marginal plots outside the two axes show histograms for the density of datapoints on each axis. Note that the ratio is frequently above 1, especially for genomes sharing between 97.5-99.5% ANI (e.g., members of different genomovars of the same phylogroup) for both species and that the estimates above ~99.5% ANI are not reliable due to inability to detect recombination at this high sequence identity level. A few outlier datapoints (genome pairs) with ratios higher than 100 were also observed in the 98-99.5% ANI range and are due to the high identity of the recombined genes identified (causing the denominator in the r/m ratio to be a small number); the majority of datapoints are between ~1 and 20. Also note that a few *E. coli* and *E. fergusoni* genome pairs (left part of the graph on the right) show a ratio higher than 1, but this is driven by recombined genes that are localized in a couple specific regions of the genome and encode specific functions (selection-driven recombination, and not widespread across the genome). See main text for additional details.

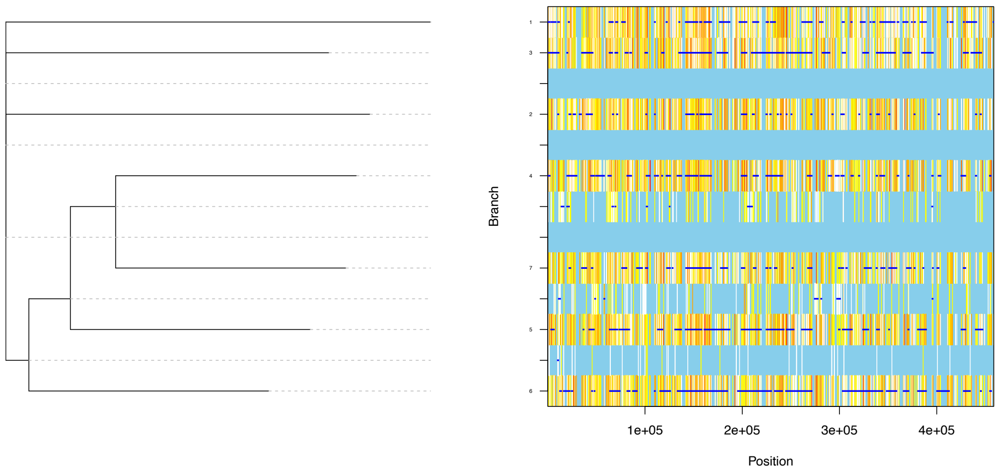

Figure S5. **ClonalFrameML analysis of SAR11 subclade Ic SAGs**. Seven SAGs with ANI>95% were used for ClonalFrameML recombination analysis (same genomes as in Figure 2a). Core genome alignment was obtained via ParSNP v1.5.3 and provided to ClonalFrameML. Each vertical line represents a SNP of the alignment, and the horizontal dark blue lines indicate strong signal for recombination for the corresponding genes. Vertical light blue indicates no substitutions; white lines represent no homoplasy while the range from yellow to orange to red reflects increasing degrees of homoplasy. The tree shown was built using the maximum likelihood method as implemented in ClonalFrameML based on the core genome alignment. Average r/m ratios were 3.82 for these SAGs vs. 1.96 for previously published *Salinibacter ruber* and 2.93 for *E. coli* genomes (the same number of genomes, with similar ANI values among the genomes, were chosen for *Salinibacter ruber* and *E. coli*) that were shown to be recombinogenic species [23]. It has been shown that ClonalFrameML generally provides similar results with several other methods when r/m ratio is less than 5 [24], as is the case here.

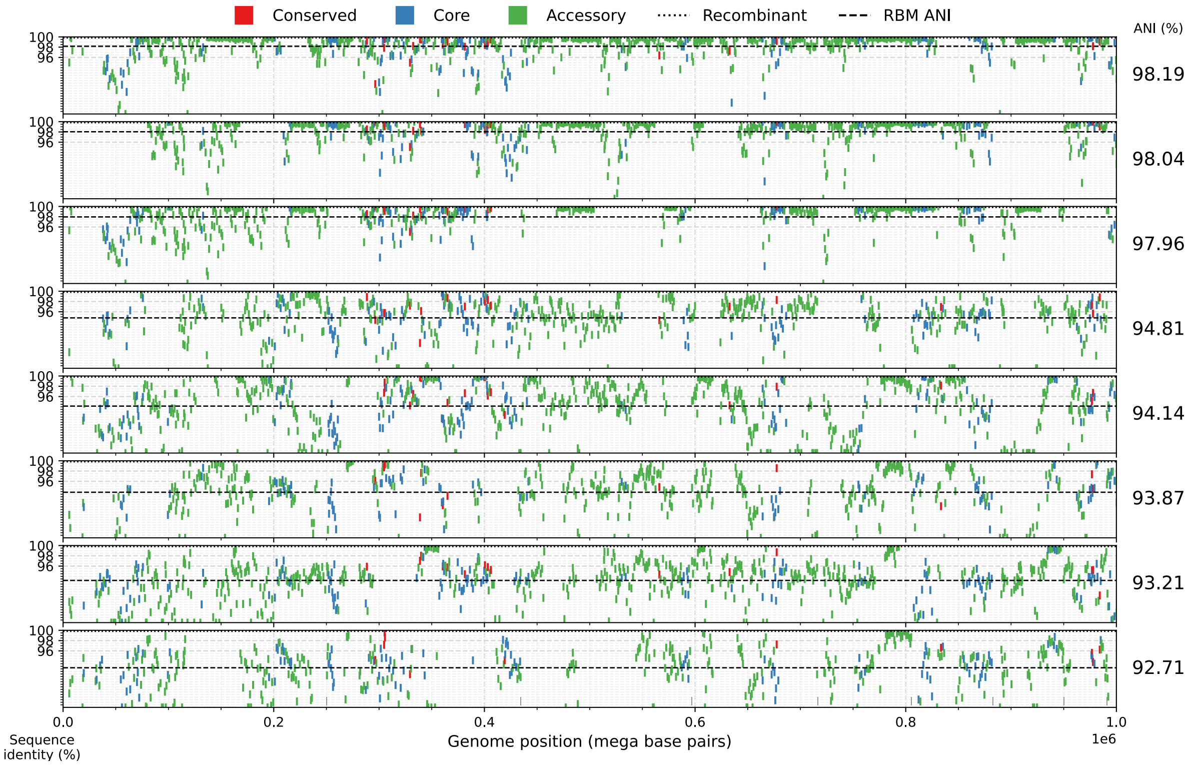

Figure S6. Extensive recent recombination within the SAR11 subclade Ib. The figure is identical to Figure 2 except that here eight SAR11 SAGs of the surface subclade Ib were compared against the same (subclade Ib) reference (AG913D08). SAGs were obtained from reference [22]. Overall, the picture of subclade Ib is remarkably similar to that of the OMZ subclade Ic (see Figure 2).

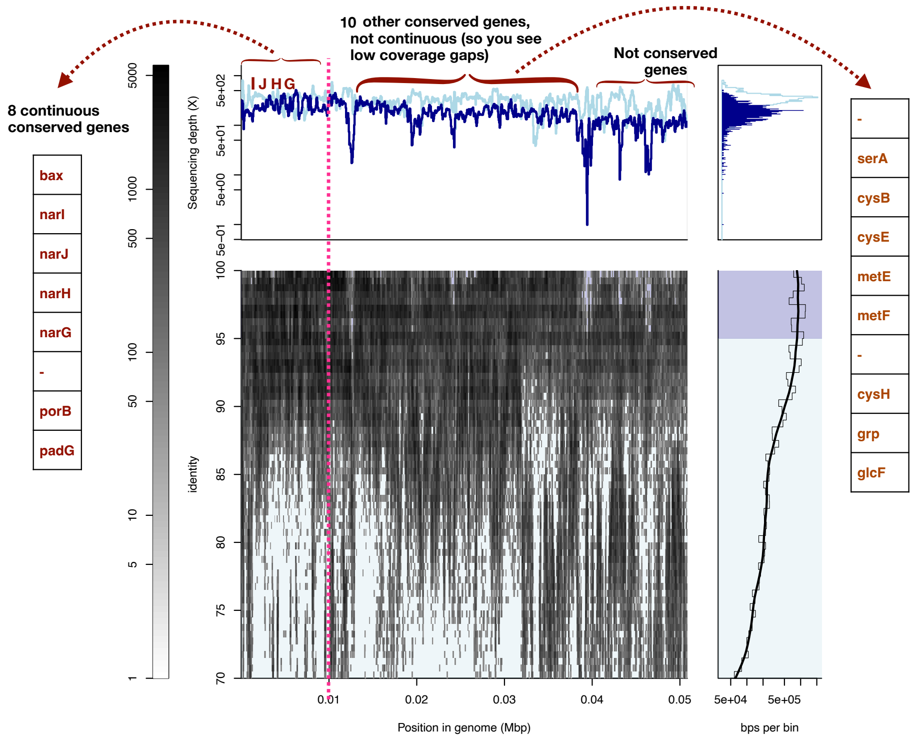

Figure S7. **Recruitment plot of the contig containing the *narG* operon**. The figure is identical to Figure S2 except that it shows only the contig that carries the *narG* operon in SAG AM-660-D08 (See Table S1 for information on the corresponding sample where this SAG originated from). This SAG was also used as the reference subclade Ic SAG in Figure 2a. Note the significantly higher identity of reads mapping to the operon relative to those mapping to downstream genes; many of the former reads likely originated from organisms that are not assigned to subclade Ic. Detailed information for the genes carried by the contig and shown on the figure can be found in Table S5.

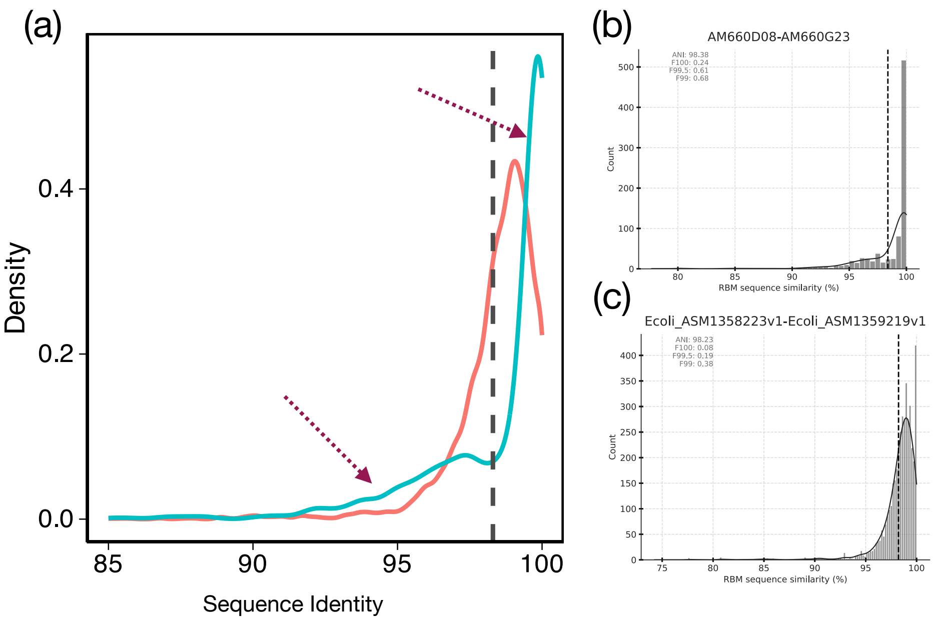

Figure S8. **Distribution of nucleotide identities of genes shared between a pair of genomes**. The reciprocal best match genes between two genomes were determined as described in the Methods section and the plots on the right show the number of shared genes (y-axis) plotted against their nucleotide identity (x-axis). The mean of each distribution is essentially the ANI value between the two genomes compared. The plot on the left (**a**) shows the same distribution but the number of genes is expressed as a fraction of total genes shared (normalized to be directly comparable between genomes of different size). The blue line represents a SAR11 SAG subclade Ic genome pair, with its underlying gene distribution shown in (**b**), whereas the red line represents an *E. coli* genome pair, with its underlying gene distribution shown in (**c**); both genome pairs showed about the same ANI, around ~98.3%. Note that the SAR11 SAGs have a significantly higher gene fraction of lower sequence identity shared genes, reflecting horizontal gene transfer mediated by recombination, from more divergent relatives of the species relative to the *E. coli* pair consistent with other results presented elsewhere.

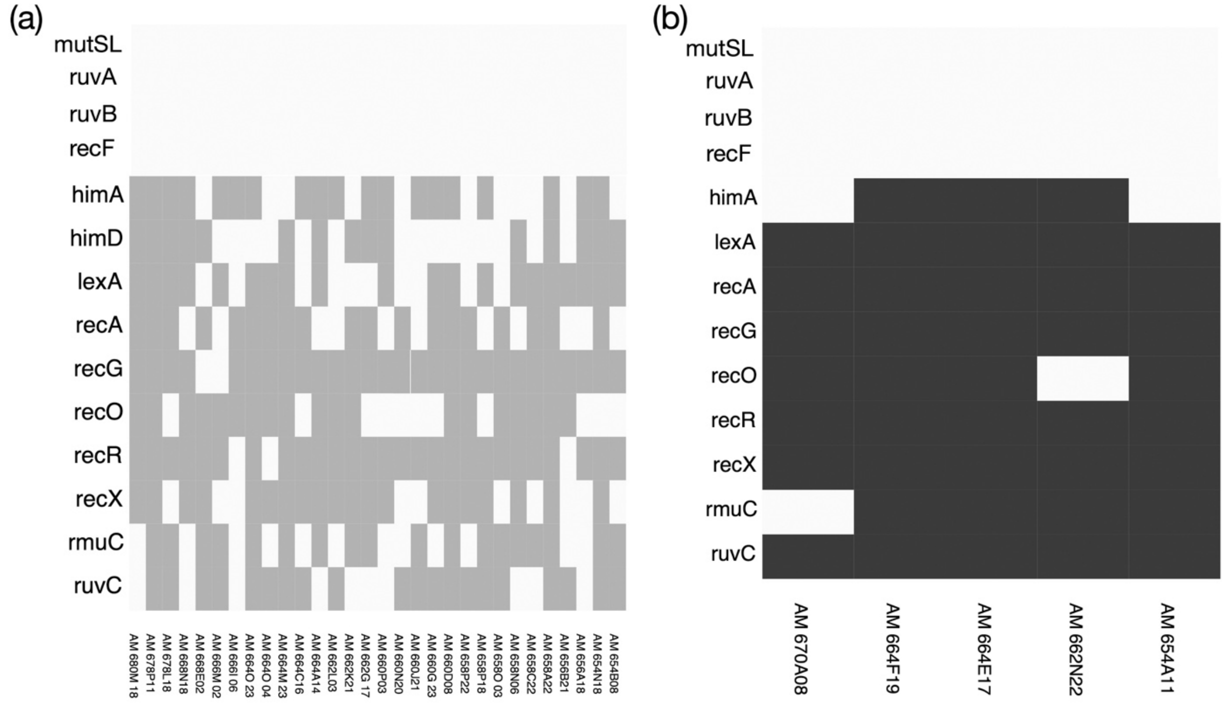

Figure S9. **Diversity in gene content related to the process of recombination among the SAG of subclades Ic (a) and IIa.A (b)**. Colored boxes denote that the corresponding gene (row) was found in the genome (column) at a minimum threshold for a match of >35% amino acid identity and >50% alignment ratio. Note that SAGs are incomplete genomes, so the fact that a gene is missing in a couple of the genomes (but is present in several others of the same subclade) is likely due to the incompleteness, although the possibility exists that the SAG truly lacks the corresponding gene. All SAGs seem to be missing the 5 genes shown on the top.

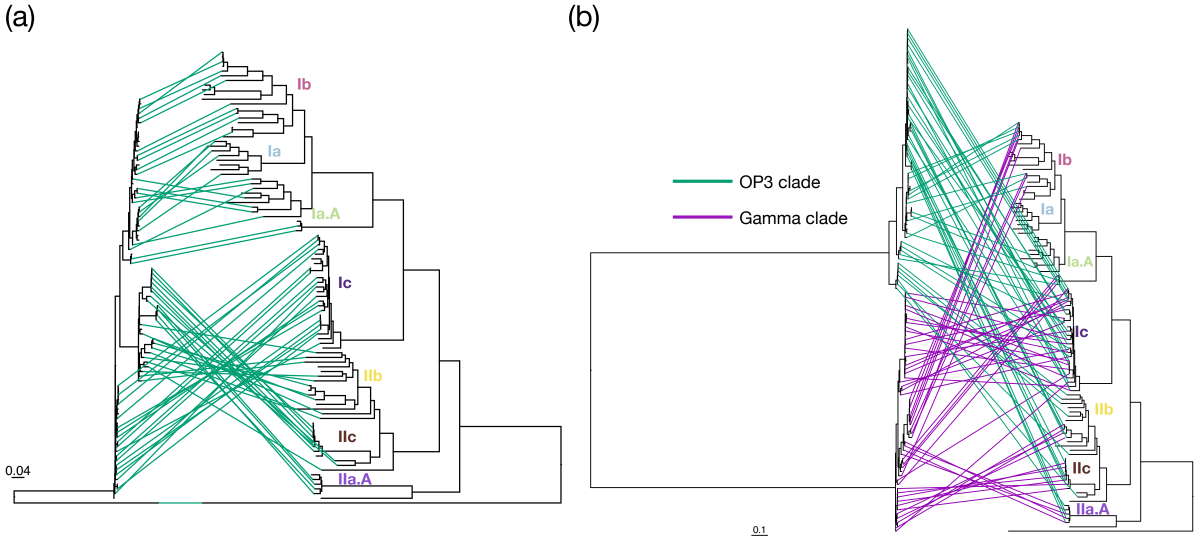

Figure S10. **Phylogenetic relationships for all SAGs based on (a) full-length 16S rRNA genes and the whole-genome and (b) NarG protein sequences and the whole genome**. The genome tree was built by extracting, aligning, trimming and concatenating amino acid sequences of universal genes from SAGs and using the resulting alignment with maximum likelihood as implemented in the GToTree software. Scale bar denotes substitution rate.

Supplementary Tables

Table S1: Sample collection location, depth and date for SAG generation and sequencing.

SAG identifier	Station	Latitude	Longitude	Depth	Collection date	O2 (umol/kg)*	Salinity (psu)	Temperature (°C)
AM-654	MEX2	18 11 N	106 17 W	80	3/11/23	0.8109	34.76	14.4
AM-656	MEX2	18 11 N	106 17 W	95	3/6/23	0.96	34.75	14.39
AM-658	MEX2	18 11 N	106 16 W	110	3/8/23	0.8429	34.79	13.81
AM-660	MEX2	18 11 N	106 17 W	130	3/11/23	0.8302	34.81	12.85
AM-662	MEX2	18 11 N	106 16 W	140	3/8/23	0.8599	34.81	12.89
AM-664	MEX2	18 11 N	106 17 W	180	3/1/23	0.858	34.79	12.25
AM-666	MEX2	18 11 N	106 16 W	400	3/8/23	0.9283	34.62	9.2
AM-668	MEX2	18 10 N	106 16 W	600	3/4/23	0.8806	34.55	7.02
AM-670	MEX2	18 10 N	106 16 W	825	3/4/23	1.2557	34.53	5.41
AM-672	MEX2	18 10 N	106 16 W	1000	3/4/23	7.573	34.54	4.56
AM-674	MEX1	18 53 N	104 54 W	40	2/25/23	9.6232	34.64	17.27
AM-676	MEX1	18 53 N	104 54 W	90	2/19/23	0.9084	34.81	14.09
AM-678	MEX1	18 53 N	104 54 W	120	2/17/23	0.8385	34.81	12.98
AM-680	MEX1	18 53 N	104 55 W	200	2/27/23	0.8856	34.78	12.17

Table S2. Sample information for the metagenomes from the ETNP OMZ used in this study.

Sample ID	DNA Concentration (µm/L)	Date Sampled	Date Extracted	Lat N	Long W	Depth (m)	Volume Filtered (mL)	Station	Oxygen Concentration (umol/kg)
1053	6.92	12/30/2021	10.16.22	12º45.662N	90º26.980W	601.1	2250	20	0.22
1055	6.16	12/30/2021	9.23.22	12º45.662N	90º26.980W	501.2	2200	20	0.06
1056	3.24	12/30/2021	10.2.22	12º45.662N	90º26.980W	451.3	2020	20	0.1
1059	3.32	12/30/2021	10.2.22	12º45.662N	90º26.980W	401.5	1950	20	0.06
1061	5.6	12/30/2021	9.23.22	12º45.662N	90º26.980W	351.5	2250	20	0.04
1063	7.32	12/30/2021	9.30.22	12º45.662N	90º26.980W	331.3	2200	20	0.07
1065	12	12/30/2021	10.12.22	12º45.662N	90º26.980W	321.6	2250	20	0.06
1067	4.52	12/30/2021	10.2.22	12º45.662N	90º26.980W	306.7	2250	20	0.36
1069	3.82	12/30/2021	9.30.22	12º45.662N	90º26.980W	251.9	2300	20	3.24
1088	4.24	01/02/2022	10.12.22	11º48.729N	94º20.501W	551.8	2100	28	0.02
1090	6.36	01/02/2022	10.12.22	11º48.729N	94º20.501W	451.9	2050	28	0.07
1092	8.72	01/02/2022	9.30.22	11º48.729N	94º20.501W	372	2150	28	0.07
1094	8.8	01/02/2022	10.16.22	11º48.729N	94º20.501W	302.1	2000	28	0.02
1096	8.36	01/02/2022	10.2.22	11º48.729N	94º20.501W	273	2160	28	0.1
1098	6.12	01/02/2022	10.2.22	11º48.729N	94º20.501W	253	2240	28	0.07
1100	5.4	01/02/2022	9.30.22	11º48.729N	94º20.501W	227	2300	28	0.75
1102	12.8	01/02/2022	10.12.22	11º48.729N	94º20.501W	173	2200	28	0.06
1104	8.32	01/02/2022	10.16.22	11º48.729N	94º20.501W	113	2160	28	1.71
1144	2.3	01/08/2022	10.2.22	12º47.379N	102º25.779W	502	2250	38	0.07
1146	7.44	01/08/2022	9.30.22	12º47.379N	102º25.779W	401	2060	38	0.07
1148	8.76	01/08/2022	10.12.22	12º47.379N	102º25.779W	352	2150	38	0.07
1150	11	01/08/2022	10.16.22	12º47.379N	102º25.779W	302	2100	38	0.05
1157	13	01/08/2022	9.30.22	12º47.379N	102º25.779W	282	2400	38	0.06
1159	4.84	01/08/2022	10.12.22	12º47.379N	102º25.779W	267	2180	38	0.49
1152	3.35	01/08/2022	9.23.22	12º47.379N	102º25.779W	202	2260	38	1.49
1154	5.6	01/08/2022	10.2.22	12º47.379N	102º25.779W	183	2340	38	0.43
1160	8.48	01/08/2022	9.30.22	12º47.379N	102º25.779W	133	2230	38	12.17
1170	4.16	01/10/2022	9.23.22	13º39.016N	103º59.970W	501	2240	40	0.07
1172	5.6	01/10/2022	9.30.22	13º39.016N	103º59.970W	402	2000	40	0.02
1174	3.98	01/10/2022	10.16.22	13º39.016N	103º59.970W	351	2220	40	0.01
1176	10	01/10/2022	10.12.22	13º39.016N	103º59.970W	301	2250	40	0.02
1178	12.3	01/10/2022	10.2.22	13º39.016N	103º59.970W	286	2380	40	0.06
1180	5.08	01/10/2022	10.2.22	13º39.016N	103º59.970W	272	2180	40	0.21
1181	4.68	01/10/2022	9.30.22	13º39.016N	103º59.970W	202	2220	40	3.11
1184	7.44	01/10/2022	10.12.22	13º39.016N	103º59.970W	147	2320	40	0.18
1186	9.4	01/10/2022	9.23.22	13º39.016N	103º59.970W	122	2600	40	0.12
1218	3.69	01/14/2022	9.30.22	16º58.790N	107º42.002W	402	2150	45	0.02
1220	4.52	01/14/2022	9.23.22	16º58.790N	107º42.002W	302	2120	45	0.04
1222	6.92	01/14/2022	10.2.22	16º58.790N	107º42.002W	252	2040	45	0.04
1224	6.8	01/14/2022	10.12.22	16º58.790N	107º42.002W	202	2120	45	0.03
1226	5.76	01/14/2022	10.2.22	16º58.790N	107º42.002W	177	2360	45	0.07
1228	10.5	01/14/2022	10.12.22	16º58.790N	107º42.002W	153	2150	45	0.03
1230	7.48	01/14/2022	9.23.22	16º58.790N	107º42.002W	132	1820	45	0.04
1231	7.88	01/14/2022	9.30.22	16º58.790N	107º42.002W	122	1900	45	0.08
1234	6.48	01/14/2022	10.16.22	16º58.790N	107º42.002W	112	2180	45	5.42

Table S3. Clade designation according to the concatenated universal gene tree in Figure 1a and quality of the 105 SAGs used in this study. "Other" denotes that there weren't enough universal genes available that can be extracted from SAGs via the GToTree software for phylogenetic placement. Genome quality was assessed using CheckM v1.5.3 and is reported on columns #3 through #5.

Genome	Clade	Completeness	Contamination	Strain_heterogeneity
AM654A08	Ia	71.55	0	0
AM656A21	Ia	52.06	1.89	100
AM656D15	Ia	74.33	0.24	0
AM674A02	Ia	67.25	0	0
AM674C22	Ia	88.73	0	0
AM674I20	Ia	76.12	0.47	0
AM674J09	Ia	75.19	0.47	0
AM674N09	Ia	73.18	0	0
AM674O03	Ia	73.66	2.41	100
AM674O10	Ia	96.21	0.47	0
AM674O15	Ia	93.84	0.95	0
AM674P21	Ia	44.77	0	0
AM670F15	Ia.A	90.77	7.23	100
AM670L02	Ia.A	59.64	0	0
AM670O04	Ia.A	82.37	0	0
AM670O07	Ia.A	52.56	0	0
AM672C17	Ia.A	74.9	7.23	100
AM672G22	Ia.A	62.98	0	0
AM672I07	Ia.A	46	0	0
AM672M07	Ia.A	79.63	0	0
AM672O05	Ia.A	72.55	0	0
AM654B03	Ib	43.98	1.2	100
AM654C02	Ib	74.86	0	0
AM654D02	Ib	70.07	0	0
AM654G16	Ib	65.25	0	0
AM658A06	Ib	44.18	1	100
AM658N19	Ib	46.23	0	0
AM664C21	Ib	71	0.71	0
AM674M03	Ib	54.2	0	0
AM678O16	Ib	58.49	0	0
AM654B08	Ic	61.87	0	0
AM654N18	Ic	74.53	0	0
AM656A18	Ic	54.76	0	0
AM656B21	Ic	59.04	0	0
AM658A22	Ic	93.8	0	0
AM658C22	Ic	46.49	0	0
AM658N06	Ic	74.36	1.2	100
AM658O03	Ic	81.64	0	0
AM658P18	Ic	68.82	0	0
AM658P22	Ic	49.23	0	0
AM660D08	Ic	82.46	0	0
AM660G23	Ic	80.16	0	0
AM660N20	Ic	55.97	0	0
AM660P03	Ic	70.25	0	0
AM662G17	Ic	59.35	0	0
AM662K21	Ic	72.96	1.2	100
AM662L03	Ic	55.19	0	0
AM664A14	Ic	61.32	0	0
AM664C16	Ic	53.48	0	0
AM664M23	Ic	53	0	0
AM664O04	Ic	55.45	0	0
AM664O23	Ic	79.66	0	0
AM666I06	Ic	49.29	0	0
AM666M02	Ic	64.95	0	0
AM668E02	Ic	84.87	0	0
AM668N18	Ic	93.98	0	0
AM678F10	Ic	60.41	0	0
AM678L18	Ic	90.47	0	0
AM678P11	Ic	85.47	0	0
AM680M18	Ic	59.75	0	0
AM654A11	Iia.A	90.1	0	0
AM662N22	IIa.A	74.92	0	0
AM664E17	IIa.A	87.37	1.53	25
AM664F19	IIa.A	76.94	0	0
AM670A08	IIa.A	87.65	0	0
AM670C11	IIb	94.98	0	0
AM670C16	IIb	94.07	0	0
AM670D10	IIb	56.56	0	0
AM670E08	IIb	78.7	0	0
AM670E14	IIb	97.59	0	0
AM670G05	IIb	71.38	0	0
AM670O10	IIb	53.63	0	0
AM670P04	IIb	71.18	0	0

AM670P07	IIb	72.09	1.2	100
AM672F18	IIb	81.91	0	0
AM672P15	IIb	82.97	0	0
AM674J13	IIb	74.23	0	0
AM674O23	IIb	87.17	0	0
AM658B22	IIc	66.36	0	0
AM662O06	IIc	94.71	0	0
AM664A04	IIc	70.08	0	0
AM664E23	IIc	60.73	0	0
AM664F23	IIc	81.53	0	0
AM664J17	IIc	69.88	0	0
AM666C10	IIc	89.74	0	0
AM654B15	Other	87.95	0	0
AM654F07	Other	87.95	0	0
AM654K14	Other	77.22	0	0
AM656C17	Other	49.95	0	0
AM656D14	Other	91.35	0	0
AM658C04	Other	63.25	1.2	100
AM658O04	Other	77.95	0	0
AM662K02	Other	78.18	0	0
AM662M16	Other	64.94	0.11	0
AM664D07	Other	80.5	0	0
AM664P01	Other	57.76	0	0
AM666F13	Other	58.66	0	0
AM668B06	Other	50.45	0	0
AM668C15	Other	77.11	0	0
AM670C09	Other	74.7	0	0
AM672N22	Other	67.91	0	0
AM680O15	Other	68.13	1.2	0

Table S4. Detailed description of the genes found in the recombination hotspot in Figure 2a (3rd red box). Gene annotation was obtained using the eggNOG-mapper v2.

Gene ID	pID(nt)	Start	Stop	Strand	COG Category	Gene Annotation	Annotation Description	Alignment Width
AM660G23_36_5	100	907436	907621	1	Hypothetical	-	Hypothetical	185
AM660G23_36_7	99.815	908243	909322	1	Metabolism 2	-	Part of the tripartite ATP-independent periplasmic (TRAP) transport system	1079
AM660G23_36_9	100	910163	910252	1	Hypothetical	-	Hypothetical	89
AM660J21_5_6	100	910252	910977	1	Metabolism 1	phbB	KR domain	725
AM660N20_19_5	99.248	911001	912197	1	Metabolism 1	phbA	Belongs to the thiolase family	1196
AM660J21_5_9	98.485	914119	915108	1	Metabolism 1	ldhA	PFAM D-isomer specific 2-hydroxyacid dehydrogenase, catalytic region	989

Table S5. Detailed description of the genes found in the recombination hotspot in Figure 2a (2nd red box). Gene annotation was obtained using the eggNOG-mapper v2. Bold fonts denote the *narG* operon.

Gene ID	pID(nt)	Start	Stop	Strand	COG Category	Gene Annotation	Annotation Description	Alignment Width
AM660G23_41_7	97.587	459999	460973	1	Conserved Hypothetical	bax	Mannosyl-glycoprotein endo-beta-N-acetylglucosaminidase	974
**AM660G23_41_6**	**98.406**	**461028**	**461720**	**-1**	**Metabolism 1**	**narI**	**Nitrate reductase gamma subunit**	**692**
**AM660G23_41_5**	**99.149**	**461717**	**462421**	**-1**	**Metabolism 1**	**narJ**	**TIGRFAM nitrate reductase molybdenum cofactor assembly chaperone**	**704**
**AM660G23_41_4**	**98.145**	**462418**	**463980**	**-1**	**Metabolism 1**	**narH**	**Respiratory nitrate reductase beta C-terminal**	**1562**
**AM660G23_41_3**	**98.81**	**463980**	**467762**	**-1**	**Metabolism 1**	**narG**	**Respiratory nitrate reductase alpha N-terminal**	**3782**
AM662K21_74_2	99.763	467791	469479	-1	Metabolism 1	-	NADPH-dependent glutamate synthase beta	1688
AM660N20_12_10	99.906	469479	470543	-1	Metabolism 1	porB	Thiamine pyrophosphate enzyme, C-terminal TPP binding domain	1064
AM660N20_12_13	99.32	472635	473516	-1	Metabolism 1	-	phosphoserine phosphatase	881
AM660N20_12_14	98.169	473506	475089	-1	Metabolism 1	serA	D-isomer specific 2-hydroxyacid dehydrogenase, catalytic domain	1583
AM662K21_34_7	97.305	476251	477252	-1	Metabolism 1	cysB	Pyridoxal-phosphate dependent enzyme	1001
AM662L03_33_7	97.631	477283	477873	-1	Metabolism 1	cysE	TIGRFAM serine O-acetyltransferase	590
AM660N20_12_21	98.596	480480	482759	-1	Metabolism 1	metE	Cobalamin-independent synthase, Catalytic domain	2279
AM660N20_12_22	98.105	482769	483665	-1	Metabolism 1	metF	Methylenetetrahydrofolate reductase	896
AM662L03_14_23	98.438	484221	484739	-1	Conserved Hypothetical	-	Protein conserved in bacteria	518
AM662K21_38_4	99.602	488277	489029	-1	Metabolism 1	cysH	Belongs to the PAPS reductase family. CysH subfamily	752
AM664M23_15_8	98.475	489351	489809	1	Information	grp	helix_turn_helix ASNC type	458
AM662L03_14_15	98.225	491656	492951	-1	Metabolism 1	glcF	4Fe-4S dicluster domain	1295

- means no gene annotation was provided from the EggNog database.

References cited.

1. Tsementzi D, Wu J, Deutsch S *et al.* SAR11 bacteria linked to ocean anoxia and nitrogen loss. *Nature*. 2016;**536**:179-83

2. Lindsay MR, D'Angelo T, Munson-McGee JH *et al.* Species-resolved, single-cell respiration rates reveal dominance of sulfate reduction in a deep continental subsurface ecosystem. *Proceedings of the National Academy of Sciences*. 2024;**121**:e2309636121

3. Bankevich A, Nurk S, Antipov D *et al.* Spades: A new genome assembly algorithm and its applications to single-cell sequencing. *Journal of computational biology*. 2012;**19**:455-77

4. Stepanauskas R, Fergusson EA, Brown J *et al.* Improved genome recovery and integrated cell-size analyses of individual uncultured microbial cells and viral particles. *Nat Commun*. 2017;**8**:84

5. Rodriguez-R LM, Gunturu S, Harvey WT *et al.* The microbial genomes atlas (MIGA) webserver: Taxonomic and gene diversity analysis of archaea and bacteria at the whole genome level. *Nucleic Acids Research*. 2018;**46**:W282-W88

6. Rodriguez-R LM, Konstantinidis KT. Nonpareil: A redundancy-based approach to assess the level of coverage in metagenomic datasets. *Bioinformatics*. 2014;**30**:629-35

7. Peng Y, Leung HC, Yiu S-M *et al.* IDBA-UD: A de novo assembler for single-cell and metagenomic sequencing data with highly uneven depth. *Bioinformatics*. 2012;**28**:1420-28

8. Rodriguez-R LM, Konstantinidis KT. The enveomics collection: A toolbox for specialized analyses of microbial genomes and metagenomes. PeerJ. 2016. https://doi.org/10.7287/peerj.preprints.1900v1.

9. Uritskiy GV, DiRuggiero J, Taylor J. Metawrap—a flexible pipeline for genome-resolved metagenomic data analysis. *Microbiome*. 2018;**6**:1-13

10. Sieber CM, Probst AJ, Sharrar A *et al.* Recovery of genomes from metagenomes via a dereplication, aggregation and scoring strategy. *Nature Microbiology*. 2018;**3**:836-43

11. Parks DH, Imelfort M, Skennerton CT *et al.* CheckM: Assessing the quality of microbial genomes recovered from isolates, single cells, and metagenomes. *Genome Research*. 2015;**25**:1043-55

12. Zhao J, Both JP, Rodriguez-R LM *et al.* GSearch: Ultra-fast and scalable genome search by combining k-mer hashing with hierarchical navigable small world graphs. *Nucleic Acids Research*. 2024:gkae609

13. Parks DH, Chuvochina M, Rinke C *et al.* GTDB: An ongoing census of bacterial and archaeal diversity through a phylogenetically consistent, rank normalized and complete genome-based taxonomy. *Nucleic Acids Research*. 2022;**50**:D785-D94

14. Altschul SF, Gish W, Miller W *et al.* Basic local alignment search tool. *Journal of molecular biology*. 1990;**215**:403-10

15. Didelot X, Wilson DJ. Clonalframeml: Efficient inference of recombination in whole bacterial genomes. *PLoS Computational Biology*. 2015;**11**:e1004041

16. Lee MD. GToTree: A user-friendly workflow for phylogenomics. *Bioinformatics*. 2019;**35**:4162-64

17. Jain C, Rodriguez RL, Phillippy AM *et al.* High throughput ani analysis of 90k prokaryotic genomes reveals clear species boundaries. *Nat Commun*. 2018;**9**:5114

18. Minh BQ, Schmidt HA, Chernomor O *et al.* Iq-tree 2: New models and efficient methods for phylogenetic inference in the genomic era. *Molecular Biology and Evolution*. 2020;**37**:1530-34

19. Edgar RC. Muscle: Multiple sequence alignment with high accuracy and high throughput. *Nucleic Acids Research*. 2004;**32**:1792-97

20. Cantalapiedra CP, Hernández-Plaza A, Letunic I *et al.* Eggnog-mapper v2: Functional annotation, orthology assignments, and domain prediction at the metagenomic scale. *Molecular Biology and Evolution*. 2021;**38**:5825-29

21. Hernández-Plaza A, Szklarczyk D, Botas J *et al.* Eggnog 6.0: Enabling comparative genomics across 12 535 organisms. *Nucleic Acids Research*. 2023;**51**:D389-D94

22. Pachiadaki MG, Brown JM, Brown J *et al.* Charting the complexity of the marine microbiome through single-cell genomics. *Cell*. 2019;**179**:1623-35. e11

23. Conrad RE, Brink, C. E., Viver, T., Rodriguez-R, L. M., Aldeguer-Riquelme, B., Hatt, J. K., Venter, S. N., Rossello-Mora, R., Amann, R., and Konstantinidis, K. T. Microbial species and intraspecies units exist 1 and are maintained by ecological cohesiveness coupled to high homologous recombination. *Nat Commun*. 2024;**15**:9906

24. Torrance EL, Burton C, Diop A *et al.* Evolution of homologous recombination rates across bacteria. *Proceedings of the National Academy of Sciences*. 2024;**121**:e2316302121
